# Climate Change and African Migrant Health

**DOI:** 10.3390/ijerph192416867

**Published:** 2022-12-15

**Authors:** Omolara Sanni, Bukola Salami, Folajinmi Oluwasina, Folakemi Ojo, Megan Kennedy

**Affiliations:** 1 Faculty of Nursing, Level 3, Edmonton Clinic Health Academy, University of Alberta, 11405 87 Avenue, Edmonton, AB T6G 1C9, Canada; 2 University of Alberta Library, University of Alberta, Edmonton, AB T6G 1C9, Canada

**Keywords:** climate change, migration, refugees, IDPs

## Abstract

Introduction: Climate change exacerbates existing sociopolitical and economic vulnerabilities, undermining livelihoods, inflating the risk of conflict, and making it difficult for people to remain stable. In 2019, around 25 million new displacements occurred due to natural disasters. This review aims to summarize the existing evidence regarding the impact of climate change on the health of African immigrants. Methods: Nine databases were systematically searched using a strategy developed in collaboration with a subject librarian. Potentially relevant articles were identified, screened, and reviewed by at least two reviewers, with a third reviewer resolving conflicts where necessary. Data were extracted from relevant articles using a standardized form. Results: Seven studies (three cross-sectional, two qualitative, one cohort, and one need assessment report) were identified; they included different categories of African migrants and reported on various aspects of health. The included articles report on climate change, e.g., flooding, drought, and excess heat, resulting in respiratory illness, mental health issues, malnutrition, and premature mortality among African immigrants. Conclusion: This review suggests climate change adversely affects the physical, mental, and social health of African immigrants. It also highlights a knowledge gap in evidence related to the impact of climate change on the health of African immigrants.

## 1. Introduction

The United Nations identifies climate change as one of the most significant crises of our time. Climate change acts as a threat multiplier, exacerbating existing sociopolitical and economic vulnerabilities, undermining livelihoods, inflating the risk of conflict, and making it difficult for people to remain stable. In 2019, approximately 50.8 million people were internally displaced across the globe, costing more than USD 4 billion a year in Africa alone [1]. In that same year, ~2 million new displacements were as a result of natural disasters [2], the occurrence of which is increasing simultaneously with climate change, related to increasing greenhouse gas concentrations, including flooding, drought, an increased frequency and intensity of climate-related disasters, and sea-level rises. UNICEF (2017) [3] predicts that, by the end of the 21st century, 70 million people and up to 30% of Africa’s coastal areas could be affected by climate change. These environmental changes are influencing human migration through their intersection with other political, economic, demographic, social, and environmental drivers of mobility [4,5]).

Climate change is associated with severe economic and social impacts and has also become a major health concern, not just for migrants. Water pollution and scarcity, increased air pollution, and changes in food production may affect anyone but, as with many stressors, climate change will hit vulnerable populations the hardest. For example, warmer temperatures are causing malaria to spread to higher altitudes [6] and will serve to increase the number of people affected worldwide. These numbers are substantial; in 2018, the World Health Organization (WHO) reported an estimated 228 million malaria episodes worldwide, 93% of which were in the WHO Africa Region [7]. Climate-related migration may have grave health effects and well-being consequences for both host and home communities. However, there is a dearth of literature on the health consequences of climate change for African migrants. This scoping review aims to identify original research articles that examine the relationship between climate change and African migrants’ health.

## 2. Methods

Our review was designed and conducted in adherence with the Preferred Reporting Items for Systematic Reviews and Meta-Analyses Extension for Scoping Reviews (PRISMA-ScR) statement [8]. This scoping review followed Arksey and O’Malley’s [9] five-stage approach.

### 2.1. Stage 1: Developing the Research Question

Our research question was: “What is known in the literature about the effect of climate change on the health of African migrants?”.

### 2.2. Stage 2: Identifying the Relevant Studies

A systematic literature search was conducted by an experienced health sciences librarian (MK) to identify all relevant published studies. Searches were performed in February 2021 using the following databases: Medline (1946–present), EMBASE (1974–present), PsycINFO (1806–present), Global Health (1910–present), and HealthSTAR (1966–present) via OVID; Cumulative Index for Nursing and Allied Health Literature (CINAHL) (1936–present) and Environment Complete (1950–present) via EBSCOhost; Scopus via Elsevier (1976–present); Sociological Abstracts (1952–present) and Dissertations and Theses Global (1861–present) via ProQuest; and Cochrane Library via Wiley (1993–present). These databases were searched using a combination of natural language vocabulary and controlled terms (subject headings) whenever available, derived from three main concepts: (1) climate change and natural disasters, (2) African migrants, and (3) health and wellness. To increase search sensitivity, publication date, language, and study type restrictions were not applied. In total, 1553 records were identified. Duplicate records (*n* = 614) were automatically removed upon import to the systematic review management software (Covidence version 2). The full search strategy is attached as an appendix.

### 2.3. Stage 3: Article Selection

**Figure 1:**
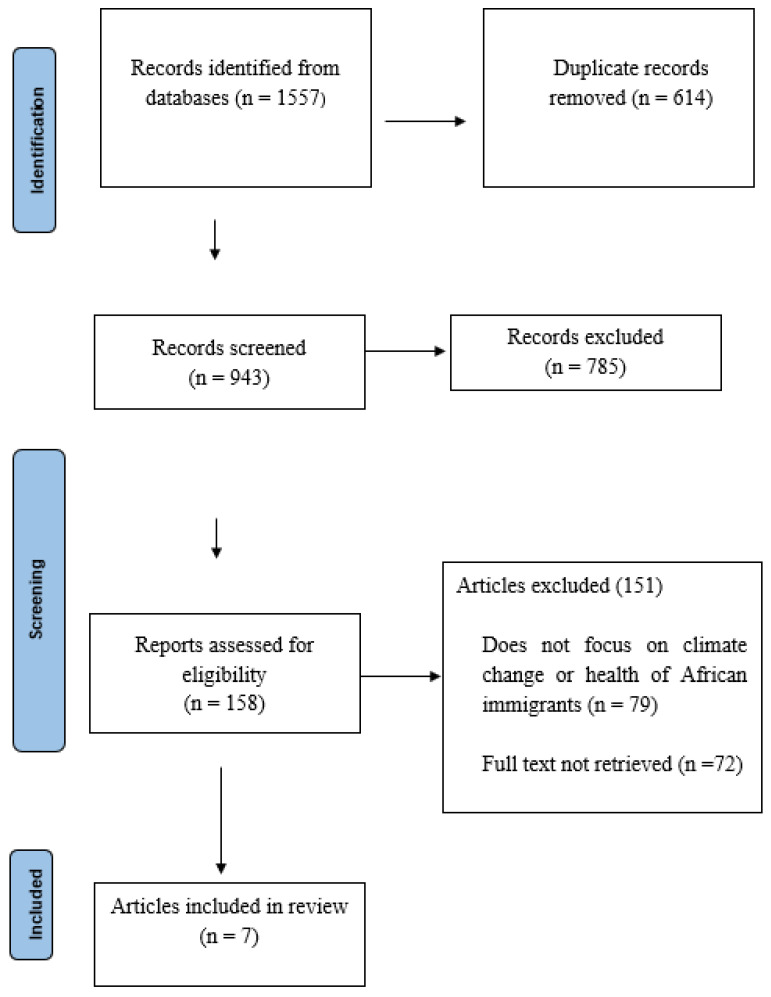
PRISMA flow diagram of article selection.

Two research personnel independently selected and reviewed each article that met the following inclusion criteria: (1) focus on climate change; (2) focus on African immigrants; and (3) focus on health. We excluded grey literature and articles that were not peer-reviewed. Disagreements were resolved by a third reviewer. A total of 943 titles and abstracts were screened for potential eligibility from which 158 articles were advanced for full-text review. Seven articles were ultimately selected for inclusion in this scoping review. We also reviewed the reference lists of included articles but found no additional studies that met our inclusion criteria. This information is summarized in detail in a PRISMA flow diagram (Figure 1).

### 2.4. Stage 4: Data Charting and Data Extraction

The following information was extracted from each of the seven articles: author(s) name, year of publication, country of study, study type, sample size, age of subjects, type of migrants, type of climate change, findings, and conclusion.

### 2.5. Stage 5: Collating, Summarizing, and Reporting the Results

The characteristics and results reported in each included article are summarily described. We present an overview of existing evidence relating to the relationship between climate change and African migrant health.

## 3. Results

### 3.1. Study Characteristics

The characteristics of the seven articles included in this scoping review are presented in Table 1. Three studies were cross-sectional, two were qualitative, one was a cohort study, and one was a need assessment report. The population size in the included articles ranged from 29 to 1.5 million. The age of participants was reported in only three studies (18–100 years, children under 5 years, and an average age of 27.9 years, respectively). Three of the seven studies included refugees, one included internally displaced persons (IDPs), one included African migrants in a European country, and one included rural-urban migrants. The included articles assessed different aspects of climate change, including flooding, exposure to particulate matter, wind speed, dew point, rainfall, and temperature, and reported on different aspects of health, including poor hygiene/ sanitation, access to healthcare, mental health, and social capital, malnutrition, respiratory health, and premature mortality. The health outcomes reported in included articles are summarized below.

### 3.2. Poor Hygiene/Sanitation

In their qualitative study focusing on refugees and IDPs, A study [14]) reports that forceful migration due to climate change often results in overcrowding because migrants tend to move from rural to urban areas. Therefore, immigrants are often faced with health issues including open defecation and a lack of or inadequate water and sanitation systems. Similarly, the authors of a cross-sectional study conducted among IDPs affected by flooding in Nigeria also suggest the significant level of sickness observed among their respondents may be due to overcrowding [10].

### 3.3. Access to Healthcare

A study [10] reported that IDPs across three states in Nigeria who were affected by the flooding had reduced access to healthcare due to damage to the infrastructure. Specifically, only 8 (57%) of the 14 pregnant women interviewed had access to antenatal care, primarily due to a lack of money to access care and transportation to the nearest facility. They also suggest the significant increase in sickness among their study population was due to a lack of access to malaria control and healthcare facilities; the proportion of IDPs in their study who had access to insecticide-treated bed nets decreased from 78.9% pre-disaster to 57.7% post-disaster. Inequities in aid provision and access to services across several refugee camps that were equally affected by drought in Kenya were reported by [11].

### 3.4. Mental Health and Social Capital

In a study by Lindvall et al. [14] identify poor mental health as one of the significant problems among IDPs and refugees affected by drought in Somalia; they report that many people were traumatized due to forced displacement as a result of war or climate change. Similarly, Heaney and Winter [13] report migrants who were displaced due to climate change in Tanzania encountered mental health issues including stress, sadness, and loneliness. In one cross-sectional study, the perception of climate change was reportedly negatively correlated with social capital (r = −0.416) among African refugees living in Nigeria, Liberia, Afghanistan, and Italy [16] that is, as the perception of climate change increased, social capital decreased. The authors also report a significant negative correlation (r = −0.256) between the loss of social capital and emotional disorders and a positive correlation between the perception of climate change and emotional disorders (r = 0.231). These findings must be interpreted with caution because a small number of non-Africans were included in the correlation coefficient calculation. Specifically, only 6 of the 100 study participants were from Afghanistan, and such a small number is unlikely to have significant effects on the reported estimates.

### 3.5. Malnutrition

A study [10] reported their study participants (IDPs affected by flooding) had significantly better feeding habits before the disaster; however, they noted that they did not consider the quality and class of the food. Lindvall et al. [14] also report that drought may lead to the loss of livestock, which consequently affects the health and nutrition of people. Dar and Khan [11] report that children in refugee camps in Kenya affected by drought suffered from severe malnutrition.

### 3.6. Respiratory Health

A cohort study that included refugees in Kenya reports a significant positive correlation between climate variables and the incidence of respiratory syncytial virus (RSV) [15]. Specifically, this included a significant moderate correlation between RSV incidence and wind speed (*p* = 0.003) and insignificant weak correlations between RSV incidence and temperature (*p* = 0.289) and RSV incidence and dew points (*p* = 0.201).

### 3.7. Premature Mortality

One article assessing the needs of IDPs suggests high rates of maternal, child, and neonatal mortality rates among persons that were internally displaced due to drought in Somalia, possibly due to shortages of trained healthcare personnel [14]. One USA study that assessed the effect of air pollution on over one million migrants from Asia, Africa, Latin America, Europe, Oceania, and North America reports that immigrants originating from Asia, Africa, and Latin America experienced higher annual average exposures to fine particles than those from other regions [12]. Although the authors did not distinguish the outcomes for African immigrants from the outcomes for immigrants from other regions, they note that the differences in average fine particle exposure were smaller between immigrants by time since immigration than by region of origin. They also report that immigrants from Asia, Africa, and Latin America had higher premature mortality attributable to fine particle exposure compared to immigrants from other regions.

## 4. Discussion

This scoping review provides an overview of existing evidence regarding the impact of climate change on the well-being of African immigrants. Findings from the articles included in this review suggest climate change may lead to a wide range of health problems in this population. Overcrowding, mental trauma, physical illness, and increased morbidity were some of the issues highlighted by the included studies as consequences of climate change across different African immigrant groups. Climate change was also reported to result in reduced access to healthcare services. Insufficient social capital and emotional disorders were associated with how refugees perceived climate change across populations.

Climate change effects such as rising temperatures, heat waves, floods, tornadoes, hurricanes, droughts, fires, deforestation, and glacier retreat, along with the disappearance of rivers and desertification, have consequences for human physical and mental health [17]. Many African groups have been displaced from their homes due to extreme weather events, resulting in homelessness and inadequate access to healthcare facilities. Unexpected and unplanned displacement due to climate change may harm people’s health; forced displacement may lead to a range of illnesses such as mental disorders and the spread of communicable diseases [7]. Additionally, inconsistent or inadequate rainfall patterns affect the supply of safe drinking water and, therefore, may lead to increases in waterborne diseases as well as drought and famine. Extreme weather conditions increase the prevalence of malnutrition and undernutrition due to plants failing to survive in unfavorable conditions. Overall, climate change is projected to lead to 250,000 additional deaths each year between 2030 and 2050 [7]. The potential impacts of climate change on the world as a whole are beginning to be better defined. However, this scoping review highlights the paucity of evidence on the effects of climate change on the health of African immigrants, hence the need for further research in this area.

## 5. Conclusions

This scoping review has a number of strengths. First, to the best of our knowledge, it is the first to provide an overview of the relationship between climate change and the health of African immigrants. Second, multiple sources were searched to identify potentially relevant articles for inclusion in the scoping review. Third, articles were thoroughly screened by at least two trained reviewers, and conflicts were resolved by a third reviewer. One major limitation of this study is the small number of articles that were included, which could limit the generalizability of the findings; as such, the results presented should be interpreted with caution. This limitation serves to highlight the need for further research in this area, especially qualitative research focusing on the lived experiences of African migrants related to climate change. Understanding how climate change affects migrants with underlying health conditions and the effect of changes in climate on maternal, child, and neonate health would be valuable, as would longitudinal studies that focus on the long-term effects of climate change on the health of African migrants.

Overall, this scoping review suggests climate change affects the physical, mental, and social aspects of African immigrants’ health. Climatic and environmental changes, population changes, and migration are strongly correlated with the development and, therefore, have a substantial impact on global sustainability discourses [18,19] and are intertwined with the effects noted here in terms of health. African migrants and IDPs are vulnerable groups, and climate change will worsen their struggle. This population is often faced with poor living conditions including overcrowded settlements, inadequate protection from weather extremes, and inadequate access to healthcare. These conditions are traumatic and underscore the stark need for mental health support services for this population. Furthermore, governments should seek to develop a long-term strategy, i.e., beyond a rapid-response system, to prevent or mitigate the health-related effects of natural disasters where possible.

## Figures and Tables

**Table 1:** Characteristics of included studies.

Authors, Year, Location	Article Type	Sample Size	Age Range (Years)	Type of Immigrants	Type of Climate Change
Amoo et al., 2018 [10], Nigeria	Cross-sectional study	432	18–100	IDPs	Flooding
Dar and Khan, 2011 [11], Kenya	Report	NR	NR	Refugees	Drought
Di Giorgi et al., 2020 [6], Multiple locations	Cross-sectional study	100	27.9 (mean)	Migrants from Nigeria, Ghana, and Cameroon	Drought
Fong et al., 2021 [12], USA	Cross-sectional study	1.5 million	NR	African immigrants	Particulate matter
Heaney and Winter, 2016 [13], Tanzania	Qualitative study	28	NR	Rural-to-urban migrants	Drought
Lindvall et al., 2020 [14], Multiple locations	Qualitative study	39	NR	Refugees and IDPs	Drought, flooding
Nyoka et al., 2017 [15], Kenya	Cohort study	NR	Children < 5	Refugees	Wind speed, dew point, rainfall, and temperature

## Data Availability

Not applicable.
